# From tensegrity to human organs-on-chips: implications for mechanobiology and mechanotherapeutics

**DOI:** 10.1042/BCJ20220303

**Published:** 2023-02-23

**Authors:** Donald E. Ingber

**Affiliations:** 1 Wyss Institute for Biologically Inspired Engineering at Harvard University, Boston, MA, U.S.A.; 2 Vascular Biology Program, Department of Surgery, Boston Children's Hospital and Harvard Medical School, Boston, MA, U.S.A.; 3 Harvard John A. Paulson School of Engineering and Applied Sciences, Cambridge, MA, U.S.A.

**Keywords:** cytoskeleton, mechanobiology, mechanotransduction, organ-on-a-chip, tensegrity, tissue engineering

## Abstract

The field of mechanobiology, which focuses on the key role that physical forces play in control of biological systems, has grown enormously over the past few decades. Here, I provide a brief personal perspective on the development of the tensegrity theory that contributed to the emergence of the mechanobiology field, the key role that crossing disciplines has played in its development, and how it has matured over time. I also describe how pursuing questions relating to mechanochemical transduction and mechanoregulation can lead to the creation of novel technologies and open paths for development of new therapeutic strategies for a broad range of diseases and disorders.

## Tensegrity and mechanobiology

My entry into the field of mechanobiology (which did not exist at that time) was unusual in that it all started in an art class. As an undergraduate student majoring in Molecular Biophysics and Biochemistry at Yale College in the 1970s, I learned that the three-dimensional (3D) shape of molecules and how they physically deform when interacting with other molecules governs how ligands activate receptors, enzymes catalyze reactions, proteins self assemble into viral capsids, and even how DNA encodes genetic information. Thus, I was intrigued when I saw art students walking around campus with sculptures they had built that looked very much like the molecules and viruses I was reading about in my textbooks. When I asked one student about the sculpture, he said that he built it in an art course called ‘Three Dimensional Design’, and suffice it to say, I did my best to get into that course.

**Figure 1.**
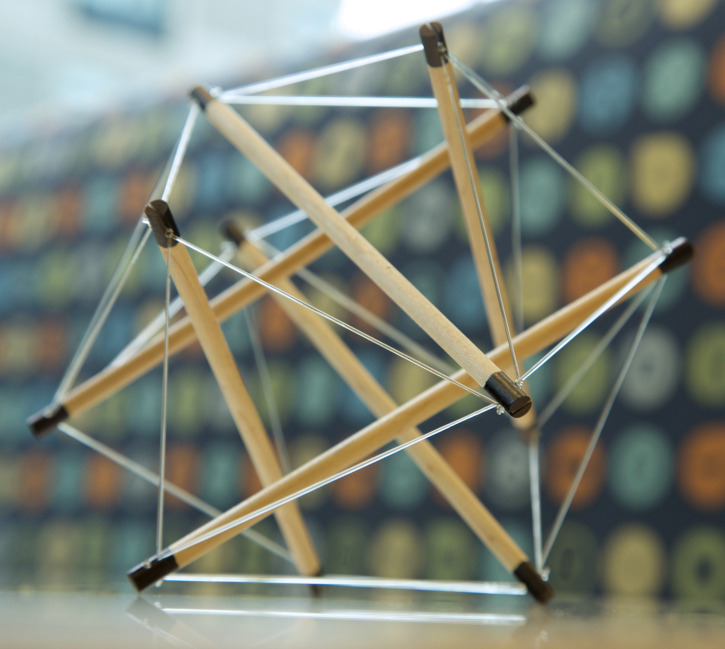
A self-stabilized tensegrity structure composed of six wood struts balanced by multiple tensed cables. This structure is similar in design to the model that the author observed in a sculpture class that flattened when pressed down and then leapt off the surface and restored itself to this rounded form upon release.

It was in this class where I first saw a tensegrity (tensional integrity) sculpture made of multiple wood dowels that did not touch each other, yet they were pulled open and stabilized in 3D by a continuous series of elastic cords, which mapped out tension field lines in geodesic (minimal distance) patterns along the edges of a rounded polyhedral form (Figure 1). When my professor pressed his hand down on this structure it flattened, and when he released it, the tensegrity spontaneously retracted and leapt up in the air taking on its rounded form once again. This amazed me because just the week before I learned how to culture cells in a cancer research laboratory at Yale School of Medicine, and when I had added trypsin to clip the cell-substrate adhesions to pass the cells from one culture dish to another, they behaved in precisely the same way. It was around this time when scientists first reported that all cells, not just muscle cells, have contractile microfilaments composed of actin and myosin in their cytoskeleton within their cytoplasm [1,2]. I also had learned that cells had other internal cytoskeletal filaments, called microtubules, which were not straight like the tensed microfilaments, but instead often displayed curved forms that suggested to me they could be buckling under compression. Thus, I immediately assumed that cells must be tensegrity structures and without knowing it, this was the beginning of my scientific career as well as the start of a resurgence of interest in the key role that mechanics plays in biology.

When I spoke of tensegrity to the postdoctoral fellow that I worked with in the cancer lab, he thought I was a bit crazy and so I decided to educate myself further before I spoke again. I started in the art library where I learned more about tensegrity and how this emerged from the work of R. Buckminster Fuller and his studies on how geodesic domes are stabilized and the seminal contributions of his student, the sculptor Kenneth Snelson. I discovered that because the cables pull in and compress the struts while the struts push out and tense the cables in tensegrities, an internal force balance is established that results an invisible state of isometric tension or tensile ‘prestress’, which physically stabilizes the 3D form of these structures much as occurs in a bow with a taught bowstring.

As I read more of Fuller's work, I learned that more complex tensegrities can be built, either as single modules with more internal elements of different sizes and shapes, or as multi-modular tensegrity assemblies. Even hierarchical structures can be built where struts and strings at one scale are themselves composed of prestressed tensegrity structures at smaller scales. Moreover, when a mechanical force is applied to one point on any of these structures, the load-bearing element does not bend or break locally; instead, the linked internal elements globally rearrange their positions and the whole structure increases its stiffness in direct response to the level of force applied. Interestingly, some artists have described the human body as a tensegrity structure as its stability depends on a balance between tensional forces generated in muscles and resisted by stiff bones, with the level of contractile tone (prestress) in muscles determining how rigid or flexible our arm, leg, neck, or torso might be.

I then moved to the biology library where I learned that at the end of the 19th century and start of the 20th (before modern biochemistry and molecular genetics had emerged), many biologists believed that mechanical forces were key regulators of biological development. I was inspired by the vision of these early scientists who focused on big questions rather than the reductionist approaches that seemed to dominate science in the late 1970s. Two of my favorite insights from early scientists came from Joseph Needham and D'Arcy Thompson. Needham wrote that ‘the problem of organization is the central problem of biology and the riddle of form is the fundamental riddle,’ while Thompson explained that ‘patterns are diagrams of underlying forces’ [3,4]. The importance of mechanical forces for control of cell form and function was also beginning to be recognized once again in the last 1970s as researchers had begun to develop ways to apply controlled mechanical forces to cultured cells and showed, for example, that mechanical stretching of embryonic muscle myotubes results in biochemical and morphological changes in cells like those observed *in vivo* during skeletal muscle hypertrophy [5].

It was during this time that I read the preface of the first edition of an early biochemistry textbook which warned the reader that for simplicity all chemical reactions in the section on thermodynamics and kinetics were assumed to take place in a well-stirred solution in a test tube. For this reason, pressure and volume terms were removed from the equations that governed these reactions. But the authors also warned that this limitation must be addressed in the future because life is clearly not a structureless chemistry. Interestingly, when I read the preface of the second and third editions of the same textbook, this warning was nowhere to be found, which in part explained why none of the other scientists around me seemed to appreciate that mechanical forces could be important. Most importantly, these observations made me realize that physical forces that alter cell shape, deform internal load-bearing structures, and change molecular conformation could indeed influence biochemistry in living cells, and that use of tensegrity architecture in the cytoskeleton might provide some insight into how this works.

The concept that underlying physical forces might guide intracellular biochemistry as well as biological pattern formation resonated with me because I had taken an undergraduate course in developmental biology where I saw some of the first time-lapse movies showing forming embryos and cultured cells under the microscope. Cells in the developing embryo seemed to pull on each other, apparently using their internal contractile cytoskeletal machinery, and this resulted in what appeared to be an almost physical sculpting of the different tissues and organs that comprise the body. Isolated epithelial cells in culture also pulled themselves flat against the rigid substrate, divided, and moved about rapidly, but they stopped when they came into contact and pulled themselves into a quiescent monolayer composed of regularly shaped polygonal cells. However, cancer cells isolated from the same tissues acted like psychotics who were oblivious to these social signals as they just kept on growing and marching right over each other. In other words, normal development appeared to be guided by mechanical forces transmitted between adherent cells, while cancer cells somehow lacked the ability to sense these physical cues.

I remained at Yale after graduation and enrolled its MD/PhD program where I carried out research to experimentally probe how cancers form and how this differs from normal tissue and organ development. In this work, I learned that tissues are not only composed of cells that adhere to each other via cell–cell junctions, they are also anchored to an extracellular matrix (ECM). The ECM is macromolecular scaffold composed of multiple types of large glycoproteins, collagens, and proteoglycans that serves to position cells and control their polarity, while also serving as both a connector and physical boundary between neighboring tissues.

As cells in tissues exhibit highly specialized forms (e.g. columnar, cuboidal, pyramidal, etc.), yet they all appear spherical when removed from tissues using enzymes that degrade these ECM molecules, I realized that this *in vivo* anchoring scaffold must normally stabilize these different cell shapes by resisting cell traction forces (due to cytoskeletal tension) much like a culture dish. In other words, even though cells exhibit stable forms when anchored to ECM in living tissues, they must exist in a state of isometric tension and experience an internal tensile prestress like a tensegrity structure. Moreover, when I was a graduate student, electron microscopists discovered that there is a close transmembrane relationship between actin microfilaments and fibronectin fibrils at sites of cell–ECM adhesion (this was termed the fibronexus before integrins and focal adhesions were discovered) [6]. To me, this observation confirmed that there must be physical coupling between these internal and external cell support scaffoldings. This meant that whole contractile cells may then serve as tensile elements that are by resisted and balanced by stiffened ECM structures (e.g. large bundles of collagen fibrils) within larger tissue and organ tensegrity structures. Indeed, surgeons know that tissues and organs are tensionally prestressed because the edges of a surgical incision spontaneously retract and must be sutured together to be held closed.

Taken together, these observations suggest that when whole organs and tissues are deformed, forces applied to the ECM will be transmitted to cells through their ECM adhesions (e.g. via integrin receptors) and from there to cytoskeletal elements and associated load-bearing molecules within the cytoplasm. Resulting stress-dependent changes in molecular shape could then alter their thermodynamic or kinetic properties locally (i.e. while not affecting nearby soluble molecules) and hence, alter intracellular biochemistry and result in force-dependent pattern formation inside living cells. Conversely, tensile forces generated within the cytoskeleton and transmitted across cell–ECM and cell–cell adhesions will induce deformations and coordinated changes in molecular structures and functions in the adjacent ECM as well as in neighboring cells and tissues. This enables long distance signaling within tissues and helps to coordinate growth and pattern formation at the cell, tissue, and organ levels.

This form of cellular ‘mechanochemical transduction’ is a central part of the tensegrity theory of cell and tissue development I proposed in which mechanical forces transmitted between cells and ECM regulate intracellular biochemistry, control cell shape and growth, and thereby guide tissue morphogenesis, whereas deregulation of this physical control system may lead to tumor formation [7–12]. This hypothesis that local differentials in mechanical forces at the cell–ECM interface drive changes in cell shape and govern normal tissue morphogenesis and that cancer formation may result through dysregulation of these processes have since been supported by results from experimental studies [13–15].

**Figure 2.**
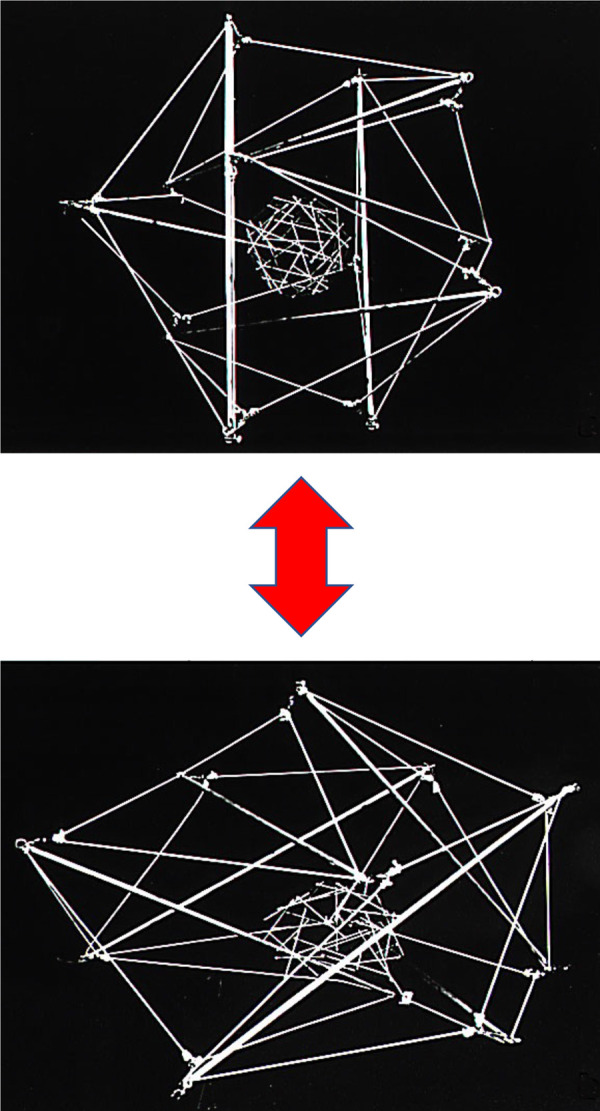
A nucleated tensegrity cell model. This model contains a larger tensegrity structure similar in design to that shown in Figure 1 and a small geodesic sphere tensegrity at its center, which are linked and prestressed by a continuous series of black tensile cables that stretch from the surface to the central structure. The black ‘cytoskeletal’ cables cannot be seen because of the black background. Note that the cell and nucleus spread in a coordinated manner when stretched and round when released (bottom vs top).

In my PhD thesis, I also constructed my own nucleated cellular tensegrity model by building a large version of tensegrity structure made of aluminum struts and bungee cords, which was linked by additional elastic strings to a smaller tensegrity sphere composed of short wood sticks and thin elastic strings that was located at its center (Figure 2). Both the cell and nucleus of this model flattened and spread out in a coordinated manner when the large model was pulled outwards and anchored to a fabric that I had attached to a rigid plate. However, when I detached the fabric's adhesions to the plate, the cell and nuclear structures spontaneously rounded once again (Figure 2) [9,10,16]. The flexible fabric also pulled up into folds, which mimicked the compression wrinkles that living cells create when they adhere and apply traction to flexible culture substrates [17]. Moreover, when the nuclear tensegrity was removed from the larger tensegrity, both structures maintained their independent shapes. All these behaviors have been observed in living cells, including the ability of nuclei and enucleated cytoplasm to maintain their respective structures and functions when isolated from each other, as well as to reconnect structurally and functionally when recombined (e.g. as in cloning of Dolly the sheep).

Thus, this experiment suggested that nucleated cells are tensegrity structures that are both multimodular and hierarchical. But what was most surprising (upsetting) to some was that these studies with this nucleated tensegrity model suggested that cell surface ECM receptors may be physically ‘hard-wired’ to the nucleus by cytoskeletal filaments. In other words, forces transmitted over transmembrane ECM adhesions could deform nuclear shape and internal chromatin structure, and thereby potentially alter gene activity directly (i.e. without requiring surface membrane signaling).

## Controversies over tensegrity and mechanotransduction

**Figure 3.**
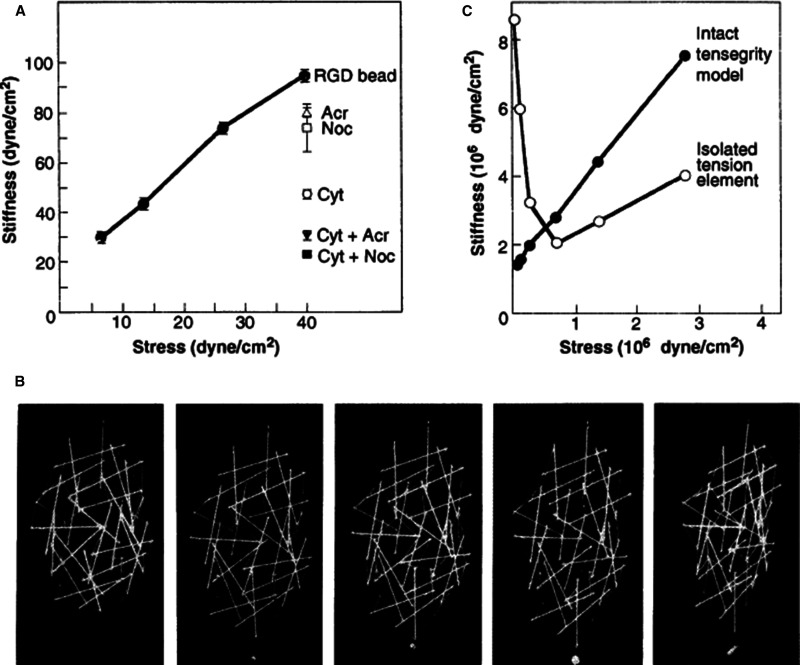
Mechanical analysis of living cells and a 3D tensegrity model. (**A**) Plot of cytoskeletal stiffness (ratio of stress to strain) as a function of applied stress measured in living cells using magnetic twisting cytometry in the absence or presence of cytochalasin D (Cyt), nocodazole (Noc) or acrylamide (Acr) to disrupt actin microfilaments, microtubules, or intermediate filaments, respectively, or combinations of these drugs. (**B**) A spherical tensegrity model built from sticks and elastic strings that was suspended from above and loaded with 0-, 20-, 50-, 100-, or 200-g weights (left to right) on a single strut at its lower end. (**C**) Stiffness of the entire tensegrity model calculated from the study shown in **B** and plotted against applied stress compared with that measured using an isolated tension element from the structure. Note that both the tensegrity structure and living cell exhibit similar linear stiffening behavior. (Reprinted with permision from ref. [24]).

The concepts that cells might be tensegrity structures, that the nucleus is hard-wired to surface receptors, and that mechanical forces may be as important as chemicals and genes were controversial from the beginning. Multiple articles attacked the theory in both scientific journals and magazines designed for the lay public [18,19], although once both sides of the argument were allowed to be presented [20,21]. As a result, I was not able to publish peer-reviewed articles that explored cellular tensegrity in detail in a mainstream journal until more than fifteen years after my original insight in the art class [16,22,23]. This was only possible after we published a breakthrough study in *Science* in 1993 that experimentally confirmed cell surface ECM receptors (integrins) behave as mechanoreceptors, that living cells and their cytoskeletons do indeed behave mechanically like tensegrity structures (e.g. exhibit linear stiffening behavior), and that interplay among all three cytoskeletal filament systems (actin microfilaments, microtubules, and intermediate filaments) contribute to this response (Figure 3) [24]. In later articles, I delved further into the use of hierarchical tensegrities in living systems and explained how this architectural principle stabilizes form at all size scales, from individual molecules, subcellular structures, organelles, and cells to tissues, organs, and whole organisms [25–27]. I also explained how tensegrity-dependent redistribution of forces in response to a mechanical stimulus can lead to mechanochemical transduction at numerous sites in the cell simultaneously, as well as how this could influence cell information processing networks and thereby control cell fate switching and development [28].

Given the wide implications of the tensegrity paradigm as well as the concept of cellular mechanotransduction, the criticisms ranged widely, but the most common ones included: (1) cytoskeletal filaments cannot bear mechanical loads because they are dynamic structures that rapidly polymerize and depolymerize, (2) there are no compression-resistant elements in cells, (3) the cytoskeleton primarily lies at the cortex beneath the surface membrane and it does not connect to the nucleus, (4) application of a local mechanical force at the surface of a cell cannot result in global structural rearrangements because of the presence of a viscous cytoplasm, and (5) transmembrane signaling is based on release of chemical signals triggered by receptor binding or clustering, and not on mechanical force transmission. One scientist even argued that cells cannot be tensegrity structures because if you cut one string in a simple tensegrity structure (e.g. 3 strut model), the entire structure loses its shape stability and this does not occur in living cells [15]. This is, of course, absurd as living tensegrities are almost always multi-modular and hierarchical (e.g. cutting the achilles tendon results in destabilization of the foot but not the rest of the body).

Over the past 40 years, these concerns have been addressed experimentally by my group and others, beginning with our demonstration that integrins are mechanoreceptors that transmit force between the cytoskeleton and ECM, and that the cell behaves mechanically like a tensegrity structure through interactions between actomyosin filaments, microtubules, and intermediate filaments when forces are applied directly to these surface receptors (Figure 3) [24]. We also experimentally confirmed that the tensile prestress in the cytoskeleton is a key determinant of cell mechanics as well as cell and nuclear form [29–31].

One of the major criticisms of the theory was based on past observations from experiments using photobleaching techniques (e.g. FRAP) with fluorescently labeled cytoskeletal protein monomers that exhibited rapid on/off rates, which suggested that all cytoskeletal filaments are constantly polymerizing and depolymerizing in living cells. However, the reality is that actin filaments and intermediate filaments are organized within larger filament bundles, much like a thick ship hawser composed of many smaller ropes. Thus, while individual monomers might be removed from and added to individual filaments at any time, the larger multi-filament structure remains intact and can still bear significant mechanical loads. We showed this directly by experimentally dissociating actin monomer turnover from the load-bearing function of actin filaments in living cells. This was done by photobleaching a local region of a contractile stress fiber containing GFP-labeled actin monomers and then using a femtosecond laser nanoscissor to physically cut through the fiber. The stress fiber exhibited a high actin turnover rate that did not change significantly after the fiber was severed and tension was dissipated [32]. This same study showed that while severing a stress fiber did not result in significant changes in positions of other actin fibers when a cell was well spread and anchored tightly to a rigid substrate. However, when a single tensed stress fiber was cut in a cell adherent to a flexible ECM gel (i.e. that more closely mimicked the material properties of a living tissue), both global rearrangements of internal cytoskeletal filaments and coordinated retraction of tensed load-bearing elements within the underlying ECM were observed.

Importantly, experimental studies also have confirmed that microtubules as well as laterally cross-linked actin filaments that create stiffer bundles (e.g. in filopodia) do indeed bear compression in living cells [31,33,34]. While individual microtubules grow and shorten, a large number of these hollow (and hence relatively stiffened) filaments organize in an aster-like structure extending from a common microtubule organizing center, and they can push out against the surrounding interconnected cytoskeletal network as well as the surface membrane. As described above, the cell's ECM anchors also can resist these cytoskeletal contractile forces and so shifting forces onto newly formed external ECM adhesions can release compressive stress on microtubules whereas depolymerizing microtubules will increase traction on the ECM [31]. This can explain why depolymerizing microtubules in a living adherent cell (e.g. cutting a major strut in a tensegrity) does not result in a total loss of cell shape stability. In addition, these complementary functions of microtubules and ECM adhesions play a key role during cell migration and neurite outgrowth as cells can extend the membrane forward via polymerization of microtubules (or actin filaments in lamellipodia and filopodia), then stabilize its new position via formation of ECM adhesions, and then reiterate the process to move or extend forward [22,35,36]. This is much like assembling a pup tent by pushing struts up against a tensed membrane that is tethered to ground anchors, then extending the strut further until the membrane is close enough to a tree branch to create a new tether above, and then releasing the strut below so that it can be repositioned and the tent reshaped.

Perhaps the most surprising result we obtained was showing that when mechanical tension is applied directly to cell surface integrin receptors that link to the cytoskeleton within focal adhesions, both cytoskeletal filaments and intranuclear structures (e.g. nucleoli) realign along the applied tension field lines [37]. Moreover, by harpooning a single nucleolus or chromosome in a cell with fine microneedle and pulling, we could demonstrate mechanical continuity within chromatin, among all chromosomes, and between these nuclear scaffolds and the surrounding cytoskeleton [38]. We now know from work done by various groups around the world that this force transfer is mediated by a series of physically linked molecules and filamentous structures that span from integrins on the surface membrane, through the cytoskeleton, and to nuclear scaffolds [39]. Interestingly, recent studies have identified a nuclear protein (LAP2b) that also mediates force transmission from the nuclear lamina to chromatin, which enables direct force-induced gene transcription inside the nucleus [40].

While recognition of the importance of tensegrity for determination of cell structure and mechanics has advanced greatly, some are still not convinced. However, the importance of integrins as mediators of mechanochemical transduction that was first predicted by my work [7,10,11] is now accepted world-wide [41]. My team has shown, for example, that applying mechanical forces directly to integrins can alter binding kinetics of linked cytoskeletal components, relocate translational machinery, and activate multiple intracellular biochemical signaling pathways (e.g. mechanosensitive ion channels, G proteins, tyrosine phosphorylation, cAMP signaling) in the cytoplasm as well as gene expression in the nucleus, whereas applying the same force to neighboring receptors (e.g. growth factor receptors) that are not physically coupled to the internal cytoskeleton does not [42–46]. Force application to surface integrins can even induce chemical signal generation deep in the cytoplasm, promote reorganization of chromatin, and turn on genes directly via force transmission across load-bearing elements inside the cell and nucleus [40,47–49]. It is important to note that because cells are surrounded by a flexible membrane and filled with viscous fluid that can dissipate mechanical energy, nature had to evolve this type of system that uses force transmission and transduction across specific molecules. However, it is possible that abnormally large-scale distortion (>40 to 100% strain) of the cell and nucleus (e.g. by being squeezed through a tiny microchannel) could bypass the need for transmembrane force transfer and result in direct deformation of cytoskeletal and intranuclear structures as well as associated biochemical changes inside the cell.

Interestingly, while distortion of the plasma membrane induced by changes in osmotic forces can also influence activities of some mechanosensitive ion channels locally at the cell surface, most of the channels located within this lipid bilayer tightly associate with the underlying tensed submembranous cytoskeleton and thus, tension in the cytoskeleton remains a key determinant of overall cell mechanics even when osmotic forces change [50]. Perhaps most importantly, numerous experiments by researchers all over the world have confirmed that this tensile prestress (level of isometric tension in the cytoskeleton), which is the fundamental hallmark of tensegrity, is a key regulator of virtually all forms of cell and tissue regulation including cell growth, motility, apoptosis, differentiation, morphogenesis, and whole organ formation [51].

Nevertheless, many scientists who model whole cell mechanics still rely on use of more conventional models that assume the cell is an elastic, viscous, or viscoelastic continuum. Others use *ad hoc* models that incorporate elastic and/or viscous support elements, which are commonly built by curve fitting experimental data. Importantly, none of these models incorporate cytoskeletal prestress which is such a critical determinant of cell mechanical behavior and they do not provide a way to link mechanical forces to specific load-bearing structures and molecules inside living cells. These other models may provide simpler and computationally more efficient solutions, but only tensegrity cell models capture the behaviors of the cell's non-linear and multi-scale structure as well as explain how changes in both mechanical and chemical signals can integrate at the molecular level.

## From tensegrity to organs-on-chips

While the emphasis on reductionism in biology led the mechanobiology field to search for ‘the’ mechanotransducer molecule, the tensegrity perspective instead suggests that the whole cell may be the mechanotransducer when it comes to functional control [27,28]. For example, many groups have observed that the overall shape of a cell — whether it is round or spread — is closely linked to its function: highly extended cells generally grow more rapidly while rounded cells remain quiescent or undergo apoptosis. However, it was assumed that these effects resulted from associated changes in chemical signaling induced by ECM receptor binding and clustering. The tensegrity theory instead suggests that these functional effects are the result of physical distortion of cell shape, which alters the internal cellular force balance, induces cytoskeletal rearrangements, and produces force-dependent changes in molecular shape and biochemical activity at many sites in the cell and nucleus almost simultaneously.

To approach this question directly, we designed an experiment in which cells are cultured on differently sized adhesive islands, each only large enough to support adhesion of a single cell. To accomplish this, we adapted a novel form of microchip manufacturing developed by George Whitesides, called soft lithography or microcontact printing. The micrometer sized islands are coated with a high density of ECM molecules to ensure optimal ECM receptor binding and are surrounded by non-adhesive regions. This way the cells spread over the ECM adhesions and then stop when they reach the non-adhesive boundary, thus causing them to take on the size and shape of the island. We also cultured the cells in a completely defined medium containing a saturating concentration of soluble growth factors.

When various types of cells were cultured on these micropatterned substrates, we found that cell shape distortion does in fact control cell fate switching. The cells progress through the cell cycle and grow when spread on large islands, cease growing and undergo apoptosis when limited to a near spherical shape and differentiate (exhibit tissue-specific functions) when held at an intermediate degree of spreading [52–54]. In addition, cells preferentially extend lamellipodia and filopodia in corner regions of square islands that promote increased local spreading relative to the sides of the islands, and hence concentrate stresses that promote focal adhesion formation in these regions [55]. We were also able to leverage this observation to engineer artificial substrates that promote directional cell movements [36]. More importantly, these publications unequivocally demonstrate that mechanical distortion of cells and changes in the cellular force balance regulate their function and control cell fate switching *in vitro*, and this was also confirmed *in vivo* in both embryonic and adult tissues [56,57].

We later modified this soft lithography fabrication approach to create microfluidic devices that are 3D structures composed of an optically clear silicone rubber material that contain hollow channels less that 1 millimeter in diameter which support fluid flow through engineered inlets and outlets. Microfluidics has been used to miniaturize various types of chemical processes, which has led to an enormous reduction in size and simplicity of analytical instruments and diagnostic devices. Working with Whitesides and his team, including a co-mentored postdoctoral fellow named Shu Takayama, we demonstrated that ECM molecules can be deposited in various patterns within these devices, which enabled living cells to be cultured, manipulated, and analyzed non-invasively under continuous fluid flow [58,59].

A few years later, after Shu had established his own independent laboratory at University of Michigan, I heard him present preliminary findings at a meeting relating to what he called a ‘Lung-on-a-Chip’, which was a microfluidic device with a hollow channel the size of a small airway in lung. There were no cells in this device at this time, but he showed that when he flowed liquid droplets through the channel to mimic mucus plugs, the device made a sound that he recorded and played for the audience, which was almost identical with the ‘crackle’ sound that I was taught to listen for through a stethoscope when I was a medical student.

I was excited by this finding and enthusiastic when Shu's student who did this work, Dan Huh, applied for a postdoctoral fellowship in my laboratory. However, when we met, I suggested that if he joined me, we should try to build a real living, breathing, lung-on-a-chip. My original idea was to flow a solution containing ECM molecules through one stream and another solution in the neighboring stream within the same microfluidic channel that would induce their polymerization and cross-linking, thereby promoting assembly of an artificial ECM scaffold at their interface. If we could accomplish this, then we could culture lung alveolar epithelial cells and microvascular endothelial cells on opposite sides of the scaffold to recreate the alveolar-capillary interface of the human lung air alveolus.

**Figure 4.**
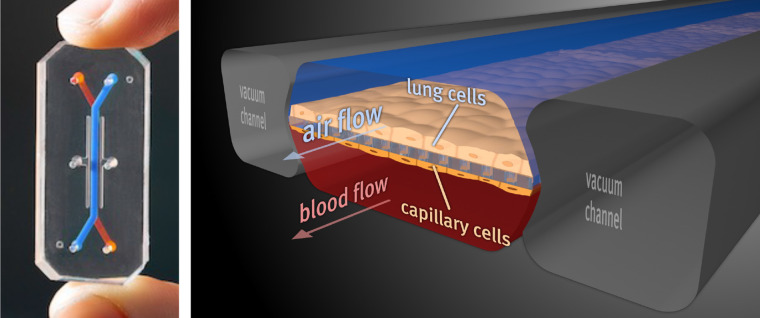
A human Lung Alveolus Chip microfluidic culture device. (Left) Photograph of a optically clear, flexible, polymeric Organ Chip containing central hollow channels for fluids and air, here highlighted with colored dye. Right) Illustration showing a cross section of the Lung Chip lined by human lung alveolar epithelial cells on the top of an ECM-coated porous membrane with human pulmonary capillary endothelial cells on the bottom of the same membrane, thereby recreating the alveolar-capillary interface. Air is introduced through the top channel to create an air-liquid interface while sustaining medium with or without immune cells is flowed through the lower vascular channel. Application of cyclic suction in hollow side chambers results in rhythmic distortion of the side walls and attached porous membrane with the adherent tissues, thereby enabling application of physiological breathing-like motions.

This turned out to be more difficult than we expected and less robust. But Dan came up with an alternative approach: he microfabricated a very thin (<50 µm thick) membrane containing multiple small (∼5 micron) pores, which he then coated with ECM and plated the different cell types on either side. This created the tissue-tissue interface we desired, but it lacked something I believed to be critical for true mimicry of the living lung microenvironment: physiological mechanical stimuli associated with cyclical breathing motions. Dan came up with a solution that involved incorporating hollow chambers on either side of the two central channels and applying cyclic suction to these compartments. When vacuum was applied, the flexible lateral walls and connected porous membrane with adherent tissues were pulled outward and when the suction was released, the stretched membrane and attached tissues retracted to their original shape. In this manner, we were able to apply cyclic mechanical deformations at the same rate and degree of distortion that living alveolar cells and tissues experience when we breathe in and out. And the first living human organ-on-a-chip (Organ Chip) with an *in vivo*-like tissue-tissue interface that experiences vascular perfusion and organ-specific mechanical cues came to life (Figure 4).

In our publication describing the breathing human Lung Chip in *Science* in 2010 [60], we demonstrated that this simple device recapitulated the organ-level structure and function of the living human lung alveolus. But more importantly, we also demonstrated that physiologically relevant mechanical cues, including both breathing motions and dynamic fluid flow, were critical to replicate *in vivo* functions with high fidelity. We and others have obtained similar results demonstrating the importance of mechanical stimulation for mimicry of human clinical responses in models of intestine, kidney, skin, bone marrow, and other organs as well. Insight into mechanical control of lung function generated using human Organ Chips has even led to the advancement of two drugs towards clinical trials [61,62].

Over the past decade, the Organ Chip field has exploded and innumerable groups around the world have confirmed the great value offered by these microfluidic models of human tissue and organ structures. Their uses include providing alternatives to animal models for drug testing as well as offering new approaches to human disease modeling, drug discovery, and personalized medicine [63]. They also provide a way to tease out contributions of multiple potential regulators of biological function — whether mechanical, chemical, molecular, or electrical — at the cell, tissue, and organ levels because each parameter can be controlled individually.

## Mechanobiology and mechanotherapeutics

While a focus on chemistry and genetics has dominated in science and medicine for decades, the reality is that there is also a strong physical basis of disease as changes in tissue structure and mechanics are often what bring a patient to the physician's office. In fact, many disorders may be considered as ‘diseases of mechanobiology’ as they share a common feature: their etiology or clinical presentation results from alterations in cell mechanics, changes in ECM structure, or dysregulation of cellular mechanotransduction [64]. This perspective also suggests that advances in mechanobiology could lead to development of new classes of mechanotherapeutics, including new drugs, improved medical devices, engineered tissues, and biologically inspired materials for tissue repair and reconstruction.

Mechanotherapies that use mechanical forces to stimulate healing or repair have been used in medicine and surgery for many years, with mechanical ventilation, distraction osteogenesis, spinal traction, orthodontics, use of tissue expanders to minimize skin defects, acupuncture, and physical therapy being simple examples [64]. There are also drugs that act by producing mechanical effects, such as bronchodilators, muscle relaxants, and ionotropic heart medications. But when it comes to mechanotherapeutic drugs that specifically target mechanotransduction, we are still early in this field. For instance, there has long been the recognition of the potential value of drugs that target mechanochemical or mechanoelectrical transducer molecules, such as cell surface mechanosensitive ion channels, for diseases ranging from cardiac arrhythmias and pulmonary edema to gastrointestinal disorders [65]. Some compounds that target these mechanosensitive channels have advanced into clinical trials. One example is the finding that breathing motions influence drug toxicities that induce pulmonary edema in the human breathing Lung Chip and that this is mediated by the mechanosensitive TRPV4 channel, which in part led to a drug that inhibits this channel being advanced into human clinical trials [61].

However, to this date, none of the drugs that target mechanosensitive channels have passed through the full clinical trial gauntlet and received FDA approval. The recent discovery of the key role that mechanosensitive Piezo channels play in various mammalian cells has raised additional excitement [66], but no new drug candidates have yet emerged. The finding that Notch, a membrane bound intercellular signaling molecule that plays a central role in differentiation and cell fate determination is also activated by mechanical forces at the cell surface [67] opens another interesting path for therapeutics development, although this has not yet been explored either.

Other drugs have been discovered that target downstream biological processes that mediate the effects of mechanical cues inside living cells. A recent example comes from another human Lung Chip study which revealed that physiological breathing motions inhibit influenza virus infections by suppressing innate immunity (i.e. increasing production of protective interferons) [62]. This was also shown to be mediated by activation of TRPV4 as well as by signaling via the receptor for advanced glycation end products (RAGE). This latter finding led to the discovery that an existing RAGE inhibitor drug (azeliragon), which had already safely passed through Phase 2 and 3 clinical trials for another medical application, could potentially be repurposed to suppress the cytokine storm triggered by some respiratory viruses. Data generated in this paper were included in an investigational new drug (IND) application submitted to the FDA to initiate a COVID-19 clinical trial by the company that manufactures the drug.

But targeting chemical signaling pathways that mediate mechanoregulation is not the only way to develop mechanotherapeutics. Altered mechanotransduction resulting from fibrotic changes in the ECM within tissue stroma, for example, can lead to a broad range of diseases from cardiac failure and pulmonary fibrosis to cancer. Thus, drugs that change ECM structure could provide a potential way to reverse disease and modulate function of cells that exhibit abnormal mechanical signaling. For instance, drugs that inhibit the collagen cross-linking enzyme lysyl oxidase (LOX) can suppress cancer growth in animal models [68]. However, this is not straightforward as there are also conflicting results which suggest that inhibition of a related ECM cross-linking enzyme (LOX-like 2) can accelerate tumor growth and decrease survival [69].

Thus, the value of targeting tissue mechanics by altering ECM cross-linking in this type of general way remains unclear. However, recent studies have revealed that synthetic ECM molecules may be administered intravenously as therapeutics that can home to their natural ECM sites to elicit repair. For example, collagen VII can be targeted to skin basement membrane to restore normal ECM structure in patients with skin diseases via intravenous injection [70]. This opens an entirely new type of mechanotherapy in which tissue mechanics may be modulated in an organ-specific way through systemic administration of engineered ECM molecules with tailored physical properties. Interestingly, as tensegrity theory predicts that ECM and intracellular microtubules have complementary load-bearing roles in cells, an alternative approach might be to target microtubules in certain medical disorders. Indeed, both inhibiting ECM cross-linking and altering microtubule polymerization have been shown to protect against heart dysfunction [71–73]. So this is an interesting future area to explore.

Perhaps the most advanced form of mechanotherapy involves drugs, such as Rho kinase (ROCK) inhibitors, that alter intracellular prestress (the central tenet of tensegrity) by inhibiting the small GTPase Rho that plays a central role in control of myosin light chain phosphorylation and cytoskeletal tension generation. ROCK inhibitor drugs, such as Ripasudil and Netarsudil, are now clinically approved for treatment of glaucoma (in Japan and United States, respectively), while Belumosudil was recently approved by the FDA for treatment of graft versus host disease. Different ROCK inhibitors are in earlier stage clinical trials or being explored in laboratories around the world for treatment of many other diseases, ranging from cerebral cavernous malformations to cardiac fibrosis.

Physical therapists have long used mechanotherapy in the form of massage, directed motion, or even acupuncture (via needle twisting) to promote musculoskeletal tissue healing via application of physical forces. However, newer and more specific forms of mechanotherapy are being developed that rely on engineered materials and flexible or ‘soft’ robotic technologies to provide physical signals necessary to trigger cellular responses required for tissue engineering or to improve function [74]. Recent work also raises the possibility that low-intensity ultrasound may provide a way to stimulate cell surface mechanosensitive ion channels and thereby achieve desired therapeutic effects non-invasively and without chemicals [75].

The clinical success of tissue engineered medical products has been limited partly because they are passive materials, their physical properties are often not optimal, and they cannot be tailored for specific applications. In response, various types of biomaterials are being developed that are engineered to provide desired mechanical stiffness or viscoelasticity necessary to provide enhanced functionality, and promising results have been obtained in preclinical models. For example, active biomaterials that can mechanically stimulate mechanosensory pathways in cells within living tissues have been developed and used to prevent muscle atrophy [76].

New materials being explored for medical applications also leverage DNA nanotechnologies that rely on molecular self assembly methodologies, such as DNA Origami, to fabricate structures with programmable material properties and 3D structures on the nanometer scale [77,78]. These structures can be modified in precise manners to display biomolecular cues (e.g. adhesive receptor ligands) along their surface in defined positions and densities with nanometer precision that can provide ways to control cell behaviors [79]. They also can be combined with peptides to create 3D hybrid scaffold materials for various medical applications, including tissue regeneration [80]. Interestingly, tensegrity principles are often used to design these self assembled DNAs to produce stable 3D structures and desired mechanical responsiveness [81,82].

Recent advances in soft robotics also have been leveraged to develop new ways to apply mechanical forces in a controlled manner to different tissues *in vivo* [74]. Implantation of magnetic ferrogels capable of massage-like compressions at sites of muscle injury have been shown to reduce fibrosis and inflammation in the injured muscle as well as enhance regeneration in an animal model [83]. A similar approach was used to create a soft robotic sleeve that fits around the beating heart and actively compresses and twists the organ to enhance cardiac ventricular function [84]. Wearable robotic devices that combine textiles and flexible materials with mechanical actuators also have been developed to apply sequential compression therapy in mobile patients with cardiovascular diseases (e.g. coronary or peripheral artery disease, deep vein thrombosis) instead of requiring patients to be immobilized, which is difficult for patients and much more expensive [85]. Critical to the design of these types of new robotic devices is the need to ensure compliance matching, that is, matching the material properties (e.g. stiffness) of the actuator to that of the natural tissues. These materials also need to move and globally rearrange their components dynamically in response to rhythmic deformations. Thus, tensegrity design principles which have already been utilized for design of free standing and moving robots [86] could be valuable here as well.

One final note brings me back to my start in molecular biochemistry and biophysics. While most biophysicists ignored my original suggestion that individual molecules use tensegrity principles to stabilize their shape [25,26], recent molecular dynamics simulations confirm that this is indeed how proteins stabilize their 3D form [87–89]. In fact, multiscale simulations reveal the use of tensegrity in individual molecules (e.g. dynein) within natural multi-molecular structures (axoneme of the sperm tail) inside living cells (whole sperm) and show that use of this hierarchical form of structural organization contributes to complex cell behaviors (sperm motility) [88]. We are now leveraging these insights to develop new computational approaches to drug design that consider intramolecular force balances and molecular context within large multiscale structures.

## Conclusion

I feel graced to have been able to contribute to the birth of mechanobiology and to see how pursuing fundamental questions and taking trans-disciplinary approaches have played such a central role in this field. The molecular and biochemical basis of mechanoregulation is increasingly well understood. Many surgeons and physicians also already accept the central importance of physical factors (e.g. breathing, peristalsis, pulse intensity, muscle contraction, tissue stiffness, etc.) when treating their patients using medical devices, surgical interventions, orthotics, or robotics. But these approaches are rarely described in mechanical terms and thus, it is important for mechanobiologists to communicate all these points more clearly in order for others to appreciate this reality. However, the bigger challenge for the future of this field is that we who pursue this path must demonstrate that mechanics is as important as chemistry and genetics for all forms of biological regulation as well as clinical care, including the development of more effective therapeutics. Only then will mechanobiology gain the broader acceptance it so well deserves.

## Abbreviations

ECM: extracellular matrix
LOX: lysyl oxidase
RAGE: receptor for advanced glycation end products
ROCK: Rho kinase
